# Work participation trajectories among 1,098,748 Finns: reasons for premature labour market exit and the incidence of sickness absence due to mental disorders and musculoskeletal diseases

**DOI:** 10.1186/s12889-019-7753-6

**Published:** 2019-10-30

**Authors:** Tea Lallukka, Erkki Kronholm, Johanna Pekkala, Sauli Jäppinen, Jenni Blomgren, Olli Pietiläinen, Eero Lahelma, Ossi Rahkonen

**Affiliations:** 1 Department of Public Health, University of Helsinki, P. O. Box 20, FIN-00014 Helsinki, Finland; 2 Finnish Institute of Occupational Health, Helsinki and Turku, Finland; 3 The Social Insurance Institution of Finland (Kela), Helsinki, Finland

**Keywords:** Sickness absence, Disability pension, Register-based, Population-based, Mental disorders, Musculoskeletal diseases, Person-oriented approach, Follow-up

## Abstract

**Background:**

Early exit from paid employment is a notable public health and societal challenge. Previous research has largely focused on the relationships among variables instead of the relationships among individuals with different work participation history. Person-oriented methods enable to identify latent groups of individuals who are likely to follow similar development in their work participation over time. We thus aimed to identify work participation trajectories during early and midlife careers and their social determinants using large nationally representative data comprising over 1 million initially employed individuals and a 10-year follow-up for their work participation. A further aim was to determine the cumulative incidence of sickness absence due to key diagnostic groups, mental disorders and musculoskeletal diseases within the trajectories.

**Methods:**

Young (25–38 years at baseline, *n* = 495,663) and midlife (39–52 years at baseline, *n* = 603,085) Finnish people, all working in 2004, were followed up through 2013, with registers of the Social Insurance Institution, and the Statistics Finland. The registers provided data for work participation and its determinants, as well as for computing the cumulative incidence of sickness absence. Latent class growth analysis was used to identify trajectories.

**Results:**

Three distinctive trajectories were identified: temporary exit, permanent exit, and continuously employed people. As compared to the other trajectories, those belonging to the permanent exit trajectory were more likely men, manual workers and had a lower income. The cumulative incidence of sickness absence due to mental disorders was highest in the permanent exit trajectory group. For musculoskeletal diseases, the cumulative incidence of sickness absence increased in the permanent exit trajectory mainly in the older age groups.

**Conclusion:**

Distinct group-based trajectories of early work exit can be identified in a representative cohort of initially employed people. Focusing on the determinants of premature exit and early intervention to tackle increasing sickness absence may promote work participation particularly in the most vulnerable groups.

## Background

Contemporary societies face a challenge and a paradoxical situation with an increasing life expectancy and a relatively short working life [1]. Although the working life expectancy has been increasing [2], a substantial part of working-age population is out of the workforce in the Western countries, and it has been estimated that the proportion of those 65 years or older will roughly double by 2050 in the World Health Organization (WHO) European region [3]. Labour market statistics further show that the proportions of people 25 years or younger, or 55 years or older in the workforce remain much lower as compared to midlife groups. Disability prevalence during working age is also high, with particularly high levels found in Finland, and other Nordic countries [4]. Given this current situation and the forecasts, there is an evident need to extend work careers from their beginning, midlife and end. Thus, to increase our understanding about how the early exit from paid employment is determined over time, it is important to identify development of work participation and its predictors during the working life span. Moreover, it is poorly understood how sociodemographic factors contribute to distinct work participation trajectories and what are the specific reasons for exit in earlier and later careers. Furthermore, previous studies have mainly examined trends in work participation using sickness absence at cross-sectional time points as in indicator of it [5, 6]. Instead, there is little evidence about the development of work participation over time using large cohort data and a nationally representative sample, following up same individuals over a longer period of time and using more comprehensive definitions of work participation, such as the inclusion of different employment statuses.

Given the high societal cost of disability during working age and early exit, a key concern of social policies is to extend work careers, and raise the retirement age [2]. To succeed in these efforts, it is important to gain novel evidence about the determinants of sustained work participation during the entire working life span, as well as pinpoint groups with a higher risk of unstable work participation or early permanent exit out of paid employment. Moreover, many studies have used small-scale survey data, have had only short follow-ups covering a minor part of working life span or narrow age groups, restricted samples (e.g. specific employee groups only), and largely variable-oriented instead of person-oriented approaches focusing thus on the relationships among variables instead of the relationships among individuals with different work participation history [7, 8]. Advanced, person-oriented methods using large scale register data enable to identify e.g. latent groups of individuals who are likely to follow similar development in their work participation over time. With these methods, it is possible to further study determinants of such development.

Musculoskeletal diseases and mental disorders are the key reasons to exit paid employment due to disability, and thus continue to be a notable health and societal burden in Finland and across many countries [4, 9–12]. It is projected that the burden of musculoskeletal and mental disorders will even continue to grow [12].

In addition to the need for novel evidence on determinants of work participation trajectories during the working life span, more evidence is needed on how such trajectories are related to ill-health e.g. the increasing incidence of sickness absence. Both musculoskeletal and mental disorders are highly prevalent and recurrent, and they increase already among young adults supporting a need to focus on their causes and consequences at an early stage [13–15]. Given that musculoskeletal diseases, particularly low back pain and neck pain strongly continue to contribute to years lived with disability [16], this further emphasizes the need to identify their risk groups during the entire working lifespan. It is equally important to focus on mental disorders, before young people become economically inactive [17], as mental ill-health affects their employment [18]. Mental disorders are strongly linked to subsequent work participation also in the later careers [19]. Overall, clear gender and age differences in both health and work participation [20], highlight the importance of assessing the predictors of work participation separately for women and men, and for younger and older employees. Such information also provides clearer policy messages, while helping identify risk groups for early exit.

Therefore, using a large register-based dataset, this study aims to produce novel evidence on work participation during working life span before statutory retirement age. More specifically, we aimed to examine early exit from paid employment, and identify work participation trajectories in two age groups, i.e. separately in earlier and later careers. This was done by using a large panel data comprising more than 1 million Finnish people, and a long follow-up covering both young employees in their early careers and midlife employees towards their later careers. Moreover, we studied sociodemographic determinants of the identified work participation trajectories, and the types of exit from paid employment by the trajectory groups. Finally, we studied differences in the cumulative incidence of sickness absence (first recorded episode during the follow-up period) due to mental disorders and musculoskeletal diseases between the identified trajectory groups, by gender and two age groups.

## Methods

The data for this study were derived from the register of the Social Insurance Institution of Finland (Kela), providing a nationally representative 70% random sample of Finnish people, who were 25 to 64 years between years 2004 through 2013. The original data constituted an unbalanced panel, which means that people could immigrate or emigrate, die, turn 25 and be included after that, or be excluded after the age of 64 years. However, the data remained representative of the Finnish working population during the entire follow-up. The data are described in more detail elsewhere [5, 21, 22].

For the purposes of this study, we included all those who were 25 to 52 years old in 2004, and were employed at the end of that year (*N* = 1,098,748). These inclusion criteria were based on several reasons as detailed below. Only all those who were employed during the baseline year, were eligible to this cohort, to be able to follow their work participation, with the baseline status of all participants being the same. All those included at 2004 were then followed up for 10 years (2004–2013) for their work participation trajectories (for operationalization, see below). The age range was selected to focus on premature exit from paid employment during working life span. Thus, at the end of the follow-up, all participants were still below 63 years, i.e., below the lowest statutory retirement age in Finland. For few occupations such as some teachers, policemen, and military officers, statutory retirement age could be lower, but such information was lacking in the available registers. Thus, the follow-up time was kept the same for all participants. As the groups would have been small, the effects on the results were likely negligible.

The data were fully register based and in addition to the data derived from the Social Insurance Institution of Finland, data from the Statistics Finland were used (socioeconomic position). The data were linked using unique personal identification numbers assigned to each citizen in Finland [23]. The participants were divided into two age groups to focus on earlier and later careers separately: 25 to 38 years (*N* = 495,663) and 39 to 52 years (*N* = 603,085) at baseline.

### Work participation

Employment status was deduced based on data on the participants’ socioeconomic position that were available for the end of each follow-up year to define exit events [24]. For each year, those who were upper or lower non-manual employees, manual workers or self-employed/farmers (altogether 10% of the population were self-employed) were classified into the working group. The rest, such as students, unemployed, those on pensions (in this age groups, mostly disability pension), those who died, and those whose position was unknown, were classified into the non-working group, i.e., formed a group which is classified as being out of paid employment either permanently (e.g. deaths) or temporarily (e.g. short-term unemployment). Employment status was dichotomized for each follow-up year separately to identify work participation trajectories in earlier and later careers. This was done to create a proxy of working-life expectancy [2], by distinguishing between the probabilities of being in the mutually exclusive statutes of either working, or not working at the end of each year. The classes in the non-working group were also examined separately, to describe in more detail the components of the exit trajectories.

### Social determinants

Baseline characteristics of the study population included gender, all taxable income, and socioeconomic position. Although some of the determinants, such as taxable income are likely time variant, for clarity all determinants were taken from the time point *before* any members of the cohort exited the paid employment. Otherwise the associations would become very complex to interpret, as a consequence, such as a change in income cannot be used to predict its cause (such as exit due to unemployment). Socioeconomic position was from the classification of the Statistics Finland [24], and when using at as a determinant of work participation trajectories, it was classified into four groups: self-employed, upper non-manual employees / white-collars, lower non-manual / white-collars and manual workers. The baseline characteristics were all considered as possible determinants of trajectory memberships.

### Sickness absence

Sickness absence was measured through medically certified sickness allowance spells, administered and paid by the Social Insurance Institution of Finland. Sickness allowance can be paid after a waiting period of ten consecutive working days of work incapacity (Sundays and midweek holidays are not counted). Related to each sickness allowance spell, the register included start and end-dates of work disability as well as the diagnosis of each spell (International Classification of Diseases, the tenth revision, ICD-10) [25]. In Finland, shorter spells are not included in national registers, and they are typically covered by the employers.

We included two key diagnostic groups for medically certified sickness absence: mental disorders (F00-F99) and musculoskeletal diseases (M00-M99), covering altogether half of all sickness absence days. We studied their cumulative incidence over the 10-year follow-up from 2004 through 2013 [25]. The cumulative incidence as indicated by the first episode of sickness absence, show differences in the incidence of sickness absence between trajectory groups among women and men and younger and midlife employees separately, by the diagnostic group.

### Ethical approval and informed consent

We used secondary register-based data. Thus, a specific ethical approval was not required. All the methods were carried out in accordance with the relevant guidelines and regulations. No informed consent was required from the participants in this fully register based study.

### Statistical analyses

We classified our study population into developmental trajectory groups describing the course of premature exit from work during a 10-year follow-up period. Individuals with at least one exit event during the follow-up made up a mixtured subpopulation where the possible number of different developmental trajectories was unknown. Therefore, we used group-based trajectory modeling (GBTM) [26] to identify possible directly unobservable developmental latent trajectories in that subpopulation. Analyses were performed by the Statistical Analysis System, SAS 9.4 Statistical Package (SAS Institute, Inc., Cary, NC) using the proc. traj procedure [27, 28]. Trajectory groups in GBTM are considered as clusters of individuals following similar developmental trajectories on an outcome over time [26]. The advantages of GBTM are e.g., the lack of need of any a priori assumption on the existence of distinct developmental trajectories. The presence of a trajectory group can be tested. The uncertainty about individuals’ group membership can be quantified in the form of probabilities [28]. For further information see e.g. Andruff et al. [29].

When determining which number of latent trajectory groups best represents the heterogeneity in developmental trajectories among the subpopulation of individuals with at least one exit event, we relied on Bayesian (BIC) and Akaike (AIC) information criteria which assess model fit by balancing model complexity versus goodness of fit [26]. Also, classification accuracy was assessed by averaging the posterior probabilities after individuals were assigned to their most likely trajectory group. In addition, in the end, the adequacy of the chosen model was considered (see, e.g. [26]). Finally, individuals without a single exit event during the whole follow-up period, i.e., who were employed at the end of each study year, were considered to form the third trajectory, as their work participation trajectory could be directly observed from the data.

After completing the classification of the whole study population, we analyzed whether the defined trajectory groups were distinguishable in terms of baseline characteristics, in other words, whether the baseline characteristics served as predictors of trajectory group memberships. Additionally, to explain more in-depth the changes in the shapes of the trajectories, we examine the reasons for exit in the identified trajectories by age. In the end, differences between trajectory groups were analyzed in terms of risk of sickness absence. Cumulative incidences of sickness absence due to mental disorders and musculoskeletal diseases were analyzed separately by gender across trajectory groups, due to established gender differences in sickness absence [30]. Cumulative incidence means that the incidence is for persons who have at least one new episode of sickness absence during the entire follow-up, irrespective of the length of that absence [31, 32]. Thus, the curves can only increase during the 10-year follow-up.

## Results

### Work participation trajectories

There were 150,147 individuals (30.3%) in the younger age group and 175,579 individuals (29.1%) in the older group who had at least one event of exit during the follow-up. The continuously employed group consisted of 345,516 individuals in the younger age group (69.7% of the group) and 427,506 in the older age group (70.9% of the group).

**Fig. 1:**
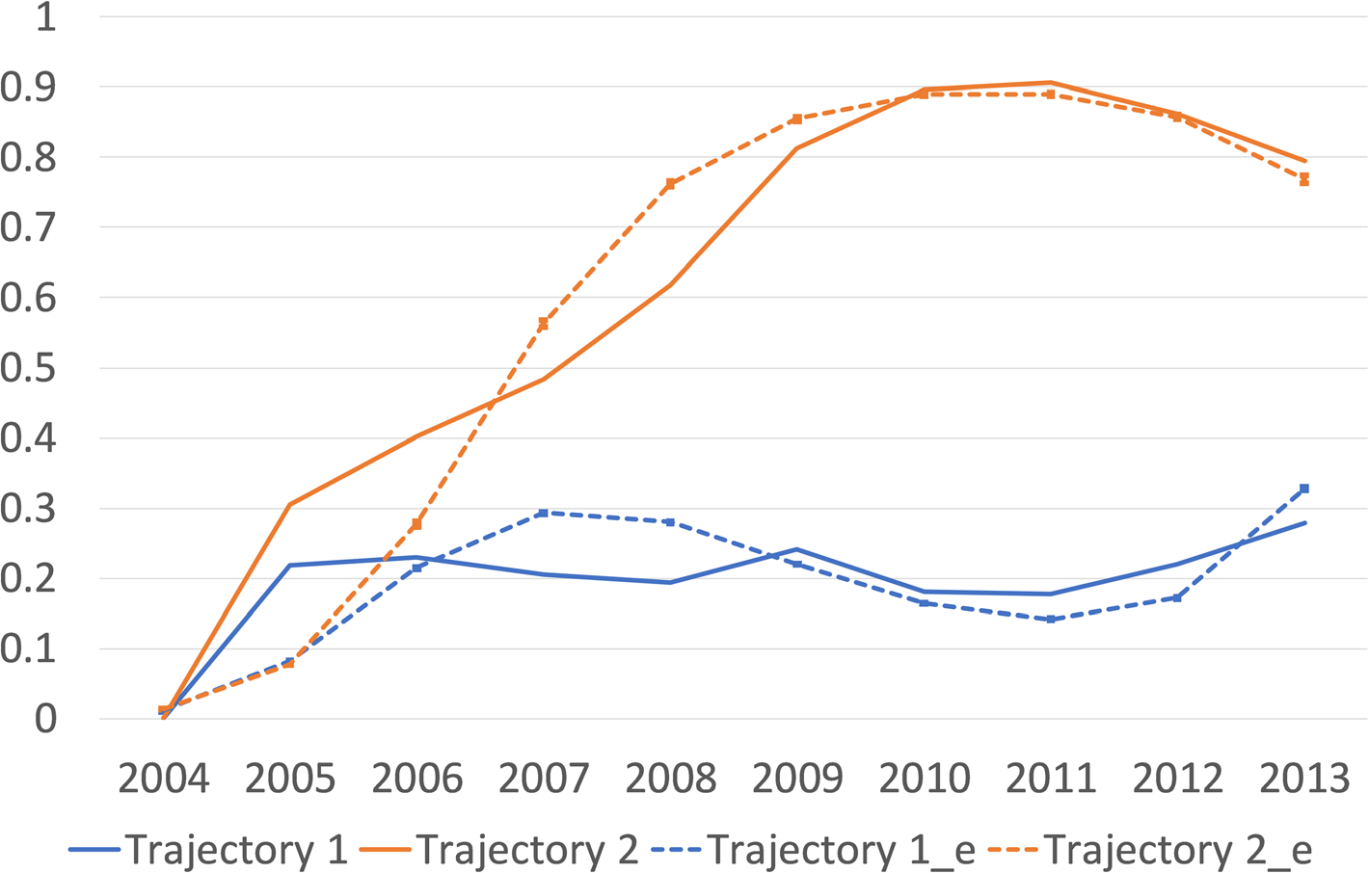
Trajectories of early labor market exit (y-axis, probability of exit) between 2004 and 2013 (x-axis) employees aged 25–38 years at baseline: Trajectory 1 = Temporary exit trajectory (83.6%), Trajectory 2 = Permanent exit trajectory (16.4%). Trajectory 1 and 2 are the averages and Trajectory 1_e and Trajectory 2_e are the model estimates with their 95% confidence intervals (not visible/very narrow due to very large numbers). Trajectory 3 = continuously employed, is on the x-axis, as their probability of exit is 0.00 throughout the follow-up

**Fig. 2:**
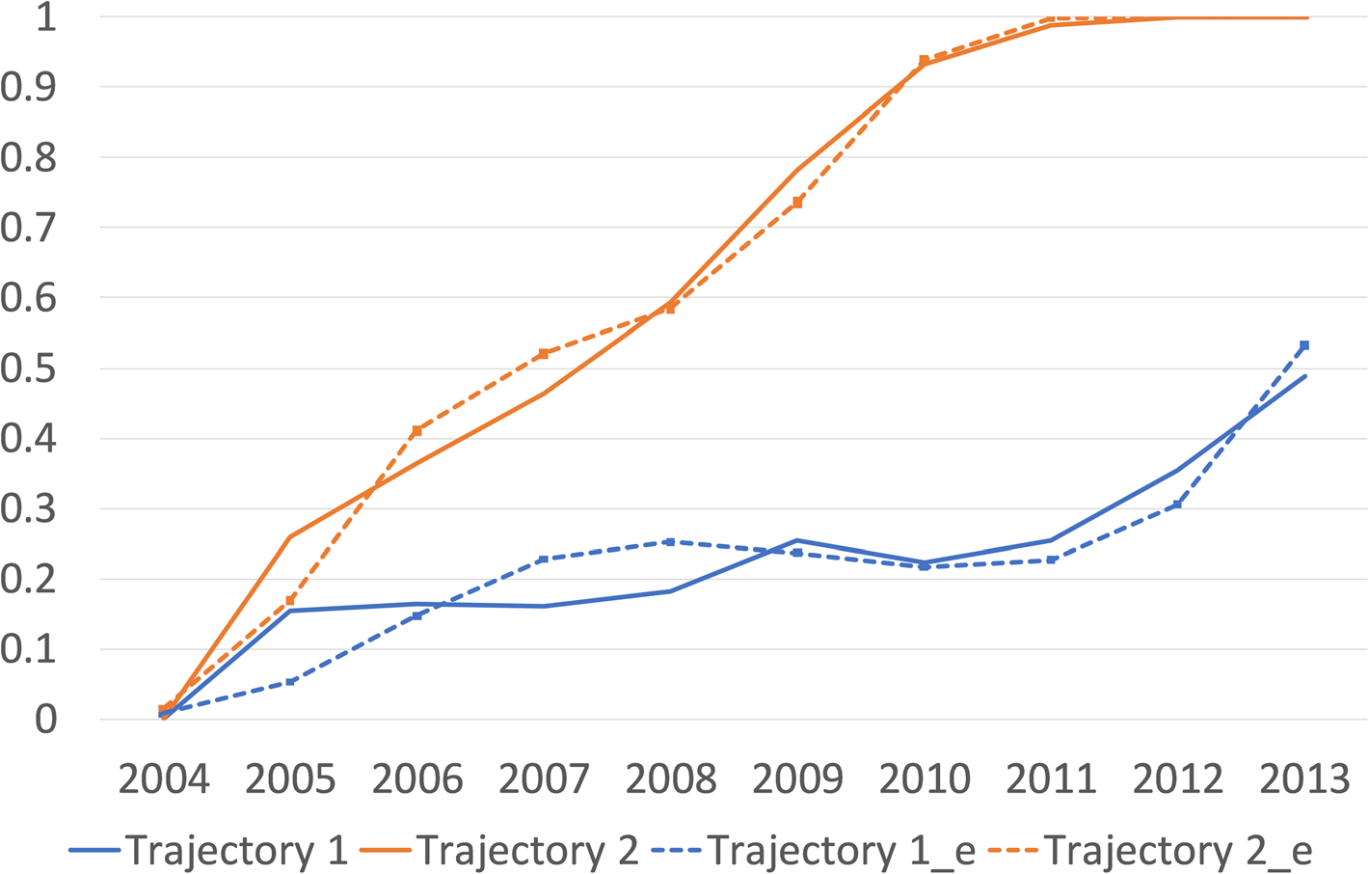
Trajectories of early labor market exit (y-axis, probability of exit) between 2004 and 2013 (x-axis) among employees aged 39–52 years at baseline: Trajectory 1 = Temporary exit trajectory (71.4%), Trajectory 2 = Permanent exit trajectory (28.6%). Trajectory 1 and 2 are the averages and Trajectory 1_e and Trajectory 2_e are the model estimates with their 95% confidence intervals (not visible/very narrow). Trajectory 3 = continuously employed, is on the x-axis, as their probability of exit is 0.00 throughout the follow-up

To determine whether the subsamples of participants with exit events could be classified into different latent trajectory groups we analyzed competing trajectory solutions of GBTM models with one and two trajectories. The solution with two trajectories (BIC = − 796,551) was clearly better than one trajectory solution (BIC = − 850,691). In these extremely large samples, solutions including also those continuously employed (whose trajectory could be more accurately and better directly observed) in the trajectory analyses, were computationally too heavy and could not be technically further considered. The two-trajectory model for younger age group is shown in Fig. 1 and for older age group in Fig. 2. The figures display first mean of the outcome which also is a probability of an event for a member of that trajectory group (as the outcome is dichotomous). Second, the predicted estimates from the model are displayed.

In both groups the two trajectories are clearly separated, the classification accuracy is good (Appendix Table 1) and they provide interpretation which is relevant for the study. In younger age-group the more prevalent trajectory (83.6% among this subgroup and 24.9% among the whole younger age group) consisted of individuals who have relatively short-term (reversible/temporary) exit events during the follow-up. The less prevalent trajectory (16.4% among this subgroup and 5.4% among the whole younger age group) consisted of individuals who have relatively long-term (non-reversible/permanent) exit events. Among the older age group the more prevalent long-term exit trajectory had the prevalence of 71.4% (among the whole older group 20.1%) and the less prevalent temporary exit trajectory had the prevalence of 28.6% (among the whole older age group 8.9%). In addition, these proportions of individuals assigned to these trajectory groups based on the posterior probability of group membership correspond closely to the estimated probability of group membership (Appendix Table 1).

In both age-groups, we then classified the study population into three developmental trajectory groups describing the course of premature exit from labor market during the 10-year follow-up period: 1) individuals with short-term reversible events of exit (temporary exit group); 2) individuals with long-term non-reversible events of exit (permanent exit group); 3) individuals without events of exit, i.e., continuously employed, serving as a reference group.

### Determinants of trajectory group memberships

Several sociodemographic determinants of trajectory group memberships were found (Table 1). Among both younger and older individuals, those belonging to the permanent exit trajectory were more likely men, manual workers and had lower income. For example, in the permanent exit trajectory, among the younger age group, those in the lowest income quartile earned less than 9300 EUR per year at baseline, whereas the cut off for the lowest income quartile among the members of continuously employed trajectory was 19,811 EUR per year in the younger age group. Men were overrepresented in the permanent exit trajectory group in both younger (55% vs. ref. 53%) and older age group (58% vs. ref. 48%). In temporary exit groups younger women were overrepresented (53% vs. ref. 47%) and older women underrepresented (47% vs ref. 52%). Among the continuously employed, the percentage men was larger in the younger age group, while in the older age group, there were slightly more women. Due to the very large data, all differences were statistically significant (*p* < 0.0001).

**Table 1:** Distributions of sociodemographic factors and number of deaths by trajectories among young (*n* = 495,663) and ageing (*n* = 603,085) employees

	Temporary exit trajectory (*n* = 123,252)	Permanent exit trajectory (*n* = 26,895)	Continuously employed over follow-up (*n* = 345,516)	*p-value/ statistical test for difference between trajectory groups*
Young employees (25–38 years)				
Men/women (%)	46.9/53.1	55.2/44.8	52.9/47.1	<.0001
Deaths	853	2267	–	.
Cut-off for the lowest income quartile (€ per year) at baseline in 2004	€13,345	€9300	€19,811	<.0001
Self-employed	5.7%	7.8%	8.3%	<.0001
Upper white-collar	18.8%	12.7%	24.0%	
Lower white-collar	34.0%	27.4%	38.5%	
Manual worker	41.5%	52.1%	29.2%	
	Temporary exit trajectory (*n* = 122,013)	Permanent trajectory (*n* = 53,566)	Continuously employed over follow-up (*n* = 427,506)	*p-value/ statistical test for difference between trajectory groups*
Ageing employees (39–52 years)				
Men/women (%)	53.3/46.7	57.6/42.4	47.7/52.3	<.0001
Deaths	3220	8684	–	.
Cut-off for the lowest income quartile (€ per year) at baseline in 2004	€18,714	€14,551	€22,531	<.0001
Self-employed	10.0%	11.6%	13.4%	<.0001
Upper white-collar	16.5%	11.6%	23.6%	
Lower white-collar	31.3%	28.0%	37.4%	
Manual workers	42.3%	48.9%	25.7%	

### Cumulative incidence of sickness absence by trajectory group

**Fig. 3:**
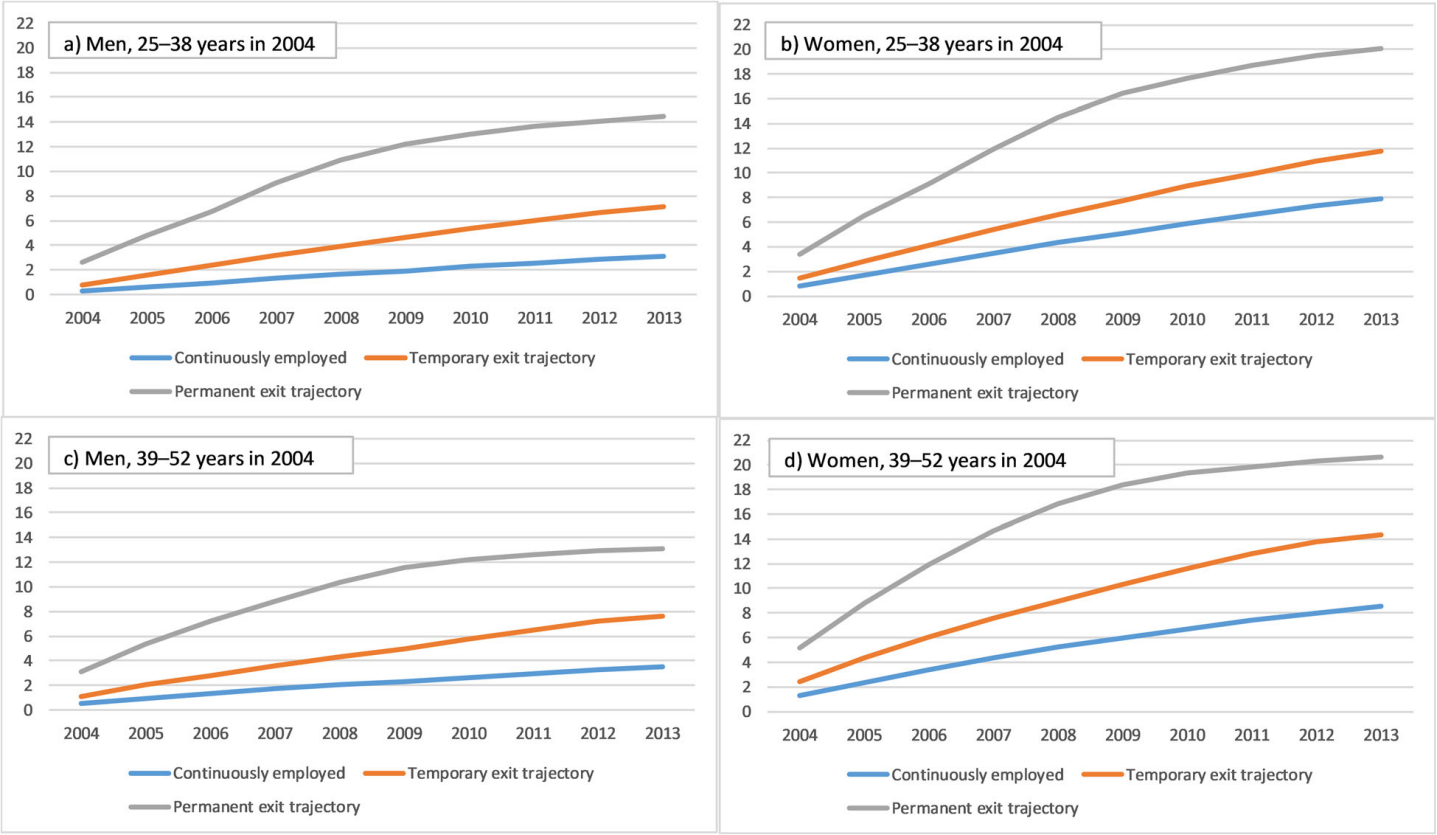
Cumulative incidence of medically certified sickness absence due to mental disorders during the follow-up (2004–2013) in three trajectory groups: Continuously employed over follow-up, temporary exit trajectory, and permanent exit trajectory. The figures are displayed for younger men (**a**), younger women (**b**), older men (**c**) and older women (**d**)

**Fig. 4:**
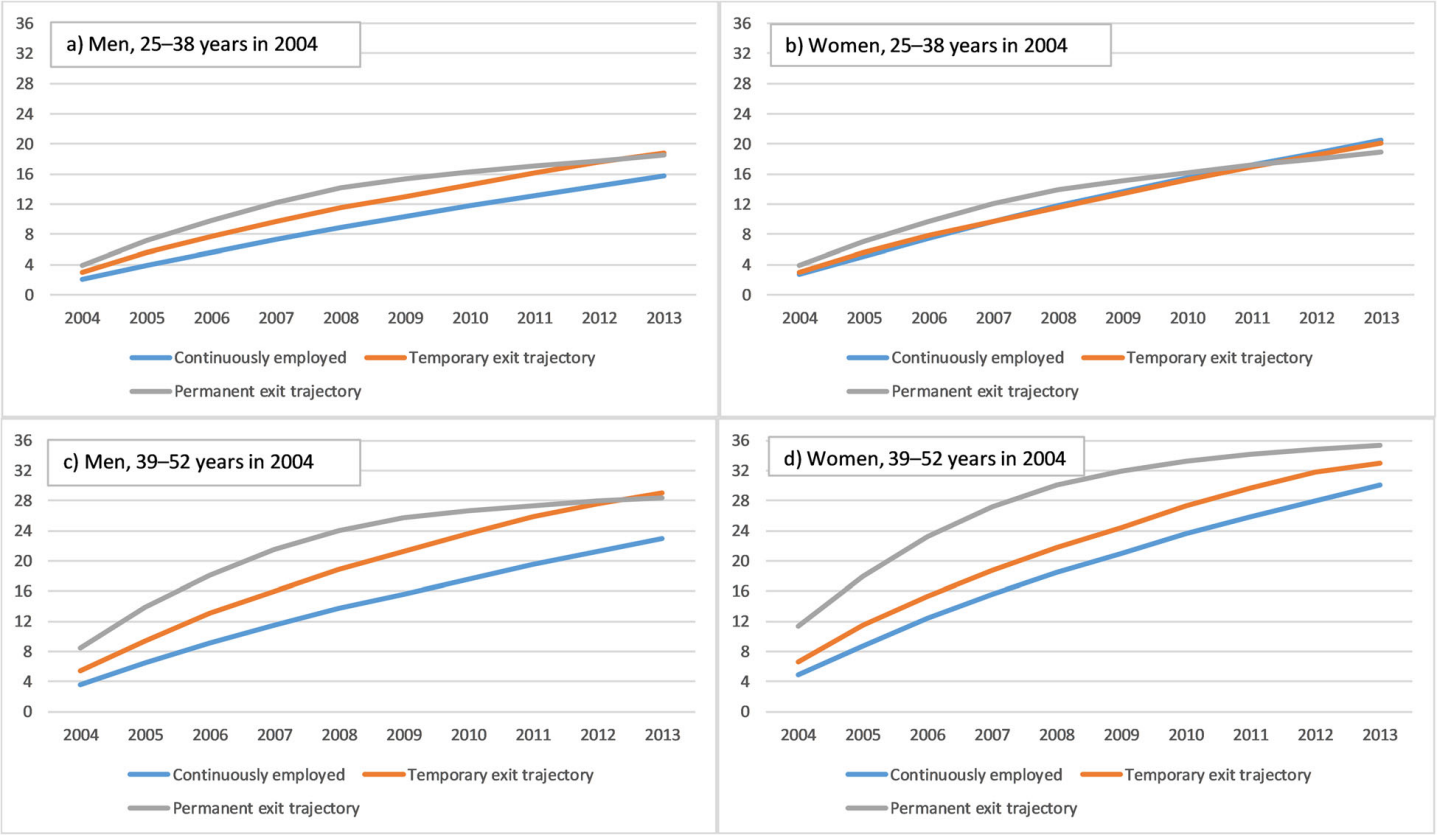
Cumulative incidence of medically certified sickness absence due to musculoskeletal diseases during the follow-up (2004–2013) in three trajectory groups: Continuously employed over follow-up, temporary exit trajectory, and permanent exit trajectory. The figures are displayed for younger men (**a**), younger women (**b**), older men (**c**) and older women (**d**)

The cumulative incidence of medically certified long-term sickness absence due to mental disorders was highest in the trajectory group of permanent exit, although the increase appeared to slow down a bit after the year 2009 and towards the end of the follow-up (Fig. 3). Regarding the other trajectories, the incidence of new events increased quite steadily throughout the 10-year follow-up. However, in absolute terms the incidence was lower in the other two trajectories, as compared to the permanent exit trajectory. In general, these patterns between trajectories were similar for women and men, and younger and older age groups. However, women’s cumulative incidence of sickness absence was higher than that of the men’s. For musculoskeletal diseases, there was little difference between the trajectory groups in the cumulative incidence of sickness absence in the young age groups, but somewhat clearer differences in the older age groups (Fig. 4). Thus, the incidence was increasing somewhat more in the temporary and permanent exit trajectory groups, as compared to the continuously employed one.

In the younger age group, there were 3120 (during the follow-up periods) deaths of which 27% took place in the temporary exit trajectory group and 73% in the permanent exit trajectory group. In the older age group there were 11,904 deaths of which also 27% took place in the temporary exit trajectory and 73% in the permanent exit trajectory (Table 1). It can be argued that to classify a death case into the temporary exit trajectory is a classification error made by the SAS proc. traj procedure. Death is obviously always a permanent exit from labor market, but the algorithm classifies some deaths which took place in the end of the follow-up period as part of the “temporary” exits. Therefore, we re-classified all death cases into the permanent exit trajectory and re-analyzed the results concerning differences between the trajectory groups across baseline characteristics and incidence rates of sickness absence. There were no meaningful differences between the original and re-analyzed results. Nonetheless, overall, the group is largely comprised of temporary type of exits, hence the name was retained as the most descriptive of the development of work participation of the members of that group.

### Reasons for exit in the trajectory groups

**Fig. 5:**
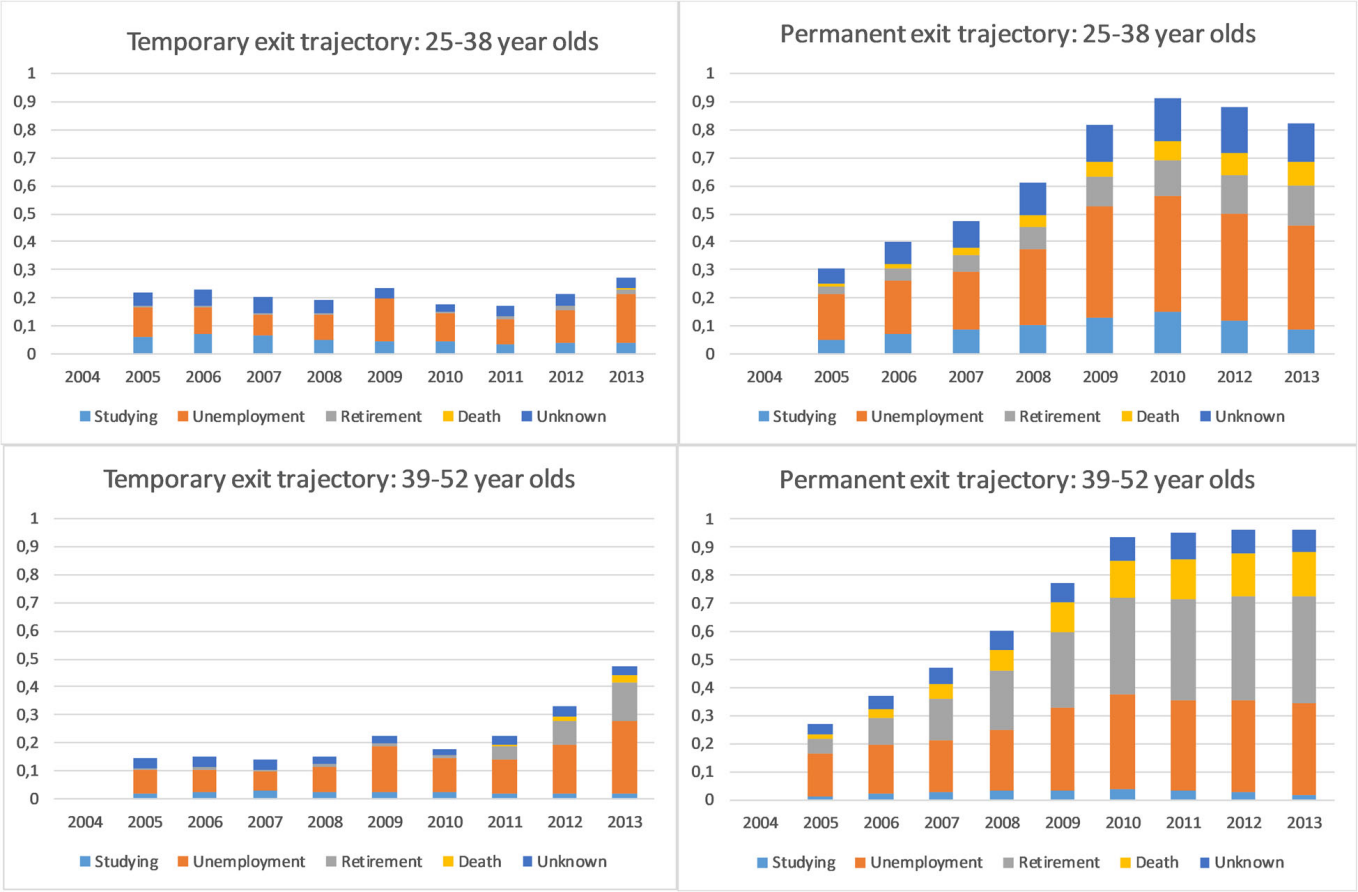
Reasons for exit (components of the exit trajectories) among younger (25–38 years) and older (39–52 years) age groups. Y-axis = probability of exit (0 = working, 1 = not working). In 2004 all were working by the cohort inclusion criteria. NB: In these age groups, retirement, i.e., the gray part of the column, is mostly disability retirement

To provide a more in-depth understanding and interpretation for the changes in the shapes of the temporary and permanent exit trajectories, we studied the specific reasons for exit in these trajectories by age (Fig. 5). The different types of exit from paid employment include studying, being unemployed, retiring, dying and other reasons. For example, Fig. 5 shows that changes in the shapes of the permanent exit trajectories among younger age group towards the end of the follow-up are linked to societal changes such as changes in unemployment rates [33]. Thus, the Fig. 5 shows that unemployment increased and was at a quite high level after the financial crisis, from 2009 onwards. Also the proportions of persons exiting the labour market due to studying had some effects on the shapes of the trajectories. Thus, in general, age affects the amount of students, but in the permanent exit trajectory, the proportions of students appeared to increase with the numbers of those unemployment until 2010, and might have decreased only when the employment situation improved. Figure 5 also displays changes in the proportions of different reasons of exit among older age group. For example, in the temporary exit group, deaths and retirements increased towards the end of the follow-up, as well as the proportions of those unemployed. This is reflected in the shape of the temporary exit trajectory towards the end of the follow-up. However, increase in the temporary exit trajectory due to deaths in the end of the follow-up could be seen as an artefact.

## Discussion

Using large nationally representative register Finnish data comprising one million younger and midlife employees with a follow-up of 10 years, we identified three distinct work participation trajectories: 1) a trajectory of individuals with temporary (reversible) events of exits; 2) trajectory of individuals with permanent (non-reversible) events of exits from labour market, and 3) a trajectory of continuously employed individuals. Sociodemographic factors (gender, taxable income, and socioeconomic position) predicted trajectory memberships both in the younger and older cohorts. There was also variation in the reasons of exit between the trajectory and age groups. Finally, trajectory memberships were associated with the cumulative incidence of sickness absence due to mental disorders in both genders and age groups and sickness absence due to musculoskeletal diseases particularly in the older cohort. The highest risk of premature exit in general and (especially) of permanent premature exit in particular was among low-income male manual workers. In all trajectory groups women had higher rates of medically certified sickness absence due to mental disorders than men. Women in the older age group had also higher rates of sickness absence due to musculoskeletal diseases than men but in the younger age-group that difference was less pronounced.

The cumulative incidence of medically certified sickness absence due to mental disorders and musculoskeletal diseases was clearly different between the trajectory groups, which is in line with previous evidence showing that people who have sickness absence have a higher risk of subsequent further sickness absence, disability pension, and unemployment, i.e., being out of paid employment due to various reasons [34–37]. Moreover, while women have more sickness absence due to mental disorders in both age groups and due to musculoskeletal diseases in the older age group, men have a higher risk of permanent exit. For young women, the higher risk to be in the temporary exit trajectory remains unclear, as our data do not enable more detailed elaboration of the reasons for their risk of exit. As people on maternity leave often have an employment, being on maternity or nursing leave is unlikely to fully explain the gender difference.

As our follow-up covered periods of economical fluctuation, it is possible that changes in sickness absence reflect simultaneous changes in unemployment, which is a cause of exit e.g. during economic downturn, leading to decreasing sickness absence [4, 38]. In contrast, during an economic upturn (until 2008), also those with poorer health are more easily employed (selection). The course and levels of sickness absence reflects also other factors besides health, i.e. people’s attitudes towards their work, work ability and health, as well as their economic situation, and other opportunities to be off work, as well as type of job and occupation they have [39]. Additionally, as numbers of people who are at risk of incident sickness absence decreases most in the permanent exit trajectory. This might partly explain why it is less likely to find any new, incident cases of sickness absence due to the examined diagnoses. In contrast, for the temporary exit trajectory, numbers who remain at risk of sickness absence remain higher, and that this could explain why the increase in sickness absence appears quite linear for the members of this trajectory. It is also of note that for self-employed and small entrepreneurs, it is more difficult to have sickness absence, (particularly if they run a very small business,) even if they were sick. This may have caused some bias in their exit events, as in the register data used, they appear to continue working. However, such groups are small and unlikely to notably affect the findings. It is further important to note that because our focus was on the differences in the *incidence* of sickness absence between trajectory groups, not the prevalence of sickness absence, this removed the effect of exit from paid employment on the estimate of sickness absence, and helped avoid complexity of causal associations and their interpretations. As permanent exit from paid employment is preceded by long-term sickness absence, prevalence figures are not meaningful, as they decrease over the follow-up giving an erroneous illustration of the development of sickness absence.

The identified work participation trajectory describing long-term or permanent exit is a clear social policy issue, since people belonging to such a trajectory are at risk of premature exit from paid employment. Further studies could aim to quantify the problems that mental disorders might cause to the members of the trajectory group. Similar attention could be paid particularly on midlife people and people towards their later careers, who have sickness absence due to musculoskeletal diseases. Moreover, further studies could estimate the risk of unemployment and the cost of sickness absence, and lost working years due to mental disorders. Since permanent exit from paid employment due to mental disorders is typically a long process, identifying people at the earliest possible stage is important, to be able to provide and target preventive measures. If mental disorders increase already years before the permanent exit events, (as indicated by the cumulative incidence of sickness absence) there could be a several years’ window of opportunity to intervene and prevent the early exit from work and working life years lost due to ill-health and work disability. Our study design does not, however, allow judging causality or temporal order between trajectory memberships and incidence of sickness absence, but these are parallel processes in these data.

Nonetheless, early exit from paid employment is a notable public health and societal challenge, particularly given the projected dramatic growth in the proportion of those over 60-year-old population in the near future [1, 3]. The results of this study have implications for health promotion and disease and disability prevention, as well as for social policies. The findings help design future interventions that are needed to understand the effects of risk factor modifications on labour market and health outcomes [40].

There are also some methodological points, and strengths and weaknesses that need to be acknowledged. Strengths of this study are the use of very large, population-based register data, representing all working aged Finnish people. Moreover, we could follow for 10 yrs for those who were employed at baseline. Administrative register data further are reliable and accurate, and none of the variables, exposures or outcomes relied on self-reported data [23]. This also helps avoid common method bias. Additionally, using more objective measures from the registers (sickness absence and disability retirement), we might have avoided systematic error due to justification for retirement, common for e.g. survey-based studies where participants may justify being out of paid employment by their perceived ill-health [41]. With large register data, it was also possible to identify trajectories in work participation separately for earlier and later careers and by gender. For women and men, the interpretations of the trajectories were the same, i.e., the shape of the trajectory did not vary by gender (measurement invariance was applied). Furthermore, we could examine determinants of work participation trajectories using register data on income and socioeconomic position, as well as examine how trajectory memberships are associated with a cumulative incidence of medically certified sickness absence due to musculoskeletal diseases or mental disorders. Thus, after identifying trajectory groups of persons that were likely to have a similar development in their work participation, these group memberships could be used to examine cumulative incidence of sickness absence.

A key limitation of this study is that due to using solely register data, several key predictors of work participation could not be included. Thus, for example health behaviors, sleep, body weight and pain can only be examined using survey data, and all these are strongly linked to the risk of work disability [42–45], and likely mediate some of the associations between social determinants and work participation trajectories. While these data are practically complete, with negligible missing data, availability of covariates in the included registers is still relatively small. In addition to health-related factors, registers lack data on working conditions, which is a limitation, since particularly physical work is linked to the risk of work disability and early exit from paid employment [46, 47]. Nonetheless, the included social factors are key predictors for both work participation and also may act as proxies for health and working conditions. Our measure of employment status also has a limitation being based on the status at the end of each year. Thus, it is possible that people who were unemployed or on a temporary disability pension or sickness absent for some period of the year were classified as continuously employed, if their state was working at the end of the year. However, in a very large data, and as the results are for the group level, such individual level misclassification is less of an issue. Our focus on the employed further provides clearer implications for preventive and other policies. Additionally, if employment status had two (or more) categories at baseline, such as included also unemployed people, the number of theoretically possible trajectories would notably increase (2^10^ = 1024), and such models would be computationally extremely heavy.

Regarding more specific reasons for exit, we further acknowledge that our method remained descriptive. However, the method was suitable for our purposes, as we aimed to give a concrete description of the reasons for exit in each age and trajectory group. Using more sophisticated statistical methods such as sequence analysis, could help produce more detailed information about the exit types within each of the trajectory groups. Such methods could be applied in future studies, extending and building on our focus and aiming to produce more policy relevant results.

Finally, it is of note that although reliability statistics were very good for the latent trajectories, they are still but approximations of true development, and some individuals can be misclassified into a latent trajectory group that does not describe their risk of work exit or developmental patterns in work participation over time. Additionally, the number of trajectories identified depends on the method used. As GBTM modelling places each participant in the trajectory group where they have the highest probability to belong to, sometimes this may be misleading in terms of interpretation. For example, it can be argued that in terms of interpretation, deaths towards the end of the follow-up should belong to the trajectory group describing permanent exit. However, the GBTM modelling placed some deaths in the short-term exit trajectory group. This was due to a short follow-up after these events. There could be also other reasons for misclassification, but as the reliabilities were high, and numbers classified with uncertain probability very low (please see Appendix Table 1), a classification error has but a negligible effect on the results. For sensitivity analyses, we also replaced all persons with a death event from the trajectory group 1 to the group 2, which due to the size of the data practically did not affect the figures in the Table 1 (data not shown) leaving the interpretation unchanged.

## Conclusion

In conclusion, distinct trajectories of early work exit can be identified in a representative cohort of initially employed people from Finland. Focusing on the determinants and reasons of premature long-term labor market exit may help promote more stable work participation particularly in the most vulnerable groups such as those with manual jobs, low income who are at particular risk of permanent exit from paid employment already during their earlier careers, and have an increasing incidence of sickness absence.

## Data Availability

The data supporting the findings of this study are available from the registered data holders (Kela and Statistics Finland) upon reasonable request, pending their permission. The data are not publicly available due to data protections laws and regulations. Thus, strict restrictions apply to the availability of these data, which were used solely under the permission from the national, administrative register data holders. The data are only available based on a specific and separate application to the register data holders, prepared according to their own guidelines, and pending their permission to access the data. Our permission number is 59/522/2015 for Kela, and TK-53-1106-15 for the Statistics Finland. The data analyzed in this study can only be accessed by persons named in the license from the register data holders.

## Appendix

**Table 2:** Prevalence of the chosen trajectories (latent class growth models, LCGM) in the latent model and after the classification based on the posterior probabilities: Temporary (T1) and permanent exit trajectory (T2) groups

	Prevalence in the model %		Prevalence after classification % (n)		Accuracy of classification (reliability = mean of the posterior probabilities, and their 95% confidence intervals)		Prevalence in the entire cohort after classification, % (n)		
Age group	T1	T2	T1	T2	T1	T2	T1	T2	T3
25–38 years	83.6	16.4	82.1 (*n* = 123,252)	17.9 (*n* = 26,895)	0.980	0.899	24.9	5.4	69.7 (*n* = 345,516)
					(0.980–0.981)	(0.897–0.901)			
Men			79.6 (*n* = 57,848)	20.4 (*n* = 14,835)	0.981	0.905	22.6	5.8	71.5 (*n* = 182,763)
					(0.980–0.981)	(0.903–0.907)			
Women			84.4 (*n* = 65,404)	15.6 (*n* = 12,060)	0.980	0.892	27.2	5.0	67.8 (*n* = 162,753)
					(0.980–0.981)	(0.889–0.894)			
39–52 years	71.4	28.6	69.5 (*n* = 122,013)	30.5 (*n* = 53,566)	0.976	0.893	20.1	8.9	70.9 (*n* = 427,506)
					(0.976–0.977)	(0.892–0.894)			
Men			67.8 (*n* = 64,979)	32.2 (*n* = 30,833)	0.975	0.893	21.7	10.3	68.0 (*n* = 203,896)
					(0.974–0.976)	(0.891–0.894)			
Women			71.5 (*n* = 57,034)	28.5 (*n* = 22,733)	0.977	0.894	18.8	7.5	73.7 (*n* = 223,610)
					(0.977–0.978)	(0.891–0.896)			

## Abbreviations

AIC: Akaike information criteria
BIC: Bayesian information criteria
GBTM: Group-based trajectory modeling
ICD-10: International Classification of Diseases, the tenth revision
Kela: Social Insurance Institution of Finland
SAS: Statistical Analysis System
WHO: World Health Organization
